# Evaluation of the college-based HIV/AIDS education policy in Beijing, China: a mixed method approach

**DOI:** 10.1186/s12199-020-00890-5

**Published:** 2020-09-10

**Authors:** Yunting Zheng, Xin Zhang, Xinying Sun, Yuhui Shi, Chun Chang

**Affiliations:** 1 Department of Social Medicine and Health Education, School of Public Health, Peking University, NO. 38 Xueyuan Road, Haidian District, Beijing, 100191 People’s Republic of China; 2 Information Management and Big Data Center, Peking University Third Hospital, Beijing, People’s Republic of China

**Keywords:** Adolescents, HIV, AIDS, Health education, Health policy, Mixed method approach, China

## Abstract

**Background:**

From 2010 to 2015, there was a twofold growth of new HIV/AIDS infection in Beijing among young students aged 15–24. HIV/AIDS education was found effective in promoting positive behavior change related to HIV/AIDS prevention. However, little evidence was found on the evaluation of HIV/AIDS education policy. This study aimed to evaluate the college-based HIV/AIDS education policy in Beijing.

**Methods:**

By using a mixed method approach, the current study reviewed college-based HIV/AIDS education policy at national level and in Beijing from 1985 to 2016 and conducted policy content analysis to evaluate the policy ability to structure implementation. Cross-sectional surveys in 2006 and 2016 were used to evaluate college’s implementation of relevant policies. *T* test, *χ*^2^ test, and logistic regression were used to analyze college students’ perception of HIV/AIDS education provided in their colleges and their knowledge of HIV/AIDS and their risk factors.

**Results:**

Fourteen pieces of national policy and four pieces of Beijing’s policy were identified. Policy’s ability to structure implementation was at moderate level. The percentage of students in Beijing who ever perceived HIV/ADIS education at colleges decreased from 71.14 to 39.80%, and the percentage of students with comprehensive knowledge of HIV/AIDS dropped from 50.00% in 2006 to 40.42% in 2016.

**Conclusions:**

HIV/AIDS education in college had drawn considerable attentions from the Chinese government, while the policy implementation needs further strengthening.

## Introduction

In 2015, all United Nations Member States adopted the 2030 Agenda for Sustainable Development with one ambitious target to end the epidemics of AIDS by 2030 [1]. In 2016, there were roughly 1.8 million new HIV infections—down from 1.9 million in 2015 [2]. However, the situation of HIV/AIDS epidemic is not that optimistic. Adolescents and young people represent a growing share of people living with HIV worldwide. In 2017 alone, 590,000 young people between the ages of 15 and 24 were newly infected with HIV, of whom 250,000 were adolescents between the ages of 15 and 19 [3]. Apart from their high risk of HIV infection, adolescents are seen as a window of hope to change toward a positive attitude and behavior which indicates investing in them is likely to be the most effective approach to confronting the epidemic.

According to the Health Development Statistics Bulletin from China’s National Health Commission [4], HIV/AIDS has become the primary cause of death among infectious diseases. For 2017, there were 57,194 cases of AIDS with the incidence rate of 4.1450/100,000 and the mortality rate of 1.1053/100,000. China’s 13th 5-year action plan for the prevention and control of AIDS [5] pointed out that sexual transmission had become the major transmission of HIV and there was a modest growth in the number of young students infected with HIV. A study used the spatial analysis to reveal the HIV/AIDS epidemic in China during 2005–2012 showed that there was a marked geographic variation of HIV infection among young people, and Beijing was identified as one of the HIV infection clustered areas [6]. From 2010 to 2015, there was a twofold growth of new HIV/AIDS infection in Beijing among young students aged 15–24 from 2.16 to 4.27% [7].

In response to the HIV/AIDS epidemic, there is sufficiently strong evidence of the effectiveness of HIV/AIDS interventions in educational settings, particularly through HIV/AIDS education interventions [8]. A review of school-based HIV interventions conducted in 2006 revealed that curriculum-based interventions incorporating key characteristics and led by adults had the strongest evidence of effectiveness and showed positive reports of behavior change [9].

Therefore, it is of great importance to understand the implementation of policies on college-based HIV/AIDS health education than to provide references for future policy development so as to achieve the SDG3.3 of ending the epidemics of AIDS by 2030. By using a mixed method approach, this study aimed to review the Chinese government’s college-based policy on HIV/AIDS health education, to analyze the ability of policy to structure implementation, and to evaluate perceived and actual policy outputs among adolescent college students in Beijing by comparing two cross-sectional survey in 2006 and 2016, respectively.

## Materials and methods

This study employed a mixed method approach using the Framework of the Implementation of Public Policy [10]. It analyzed the ability of college-based HIV/AIDS health education policy to structure implementation and the target group-adolescent college students’ perceived impact of policy outputs and the actual impacts of policy outputs.

### Policy search

College-based HIV/AIDS education policies were searched through websites of following Chinese government website:

- State Council, http://english.gov.cn/policies
- National Health & Family Planning Commission (NHFPC), http://en.nhfpc.gov.cn/
- Ministry of Education, http://en.moe.gov.cn/
- Beijing government, http://www.ebeijing.gov.cn/
- Beijing Municipal Education Commission, http://jw.beijing.gov.cn/
- Beijing Municipal Commission of Health and Family Planning, http://wjw.beijing.gov.cn/english/

### Policy inclusion and exclusion criteria

This study used a modified PICOT (Population, Intervention, Comparison, Outcome, and Time) format [11] to summarize the inclusion criteria of policy review by replacing the *Comparison* with the *Setting*. The inclusion criteria of policy review were described as below:

- Population: college students
- Intervention: HIV/AIDS health education
- Setting: colleges in People’s Republic of China
- Outcome: national policy + policy in Beijing
- Time period: 1985–2016

Exclusion criteria were structured using the same format:

- Population: non-college students
- Intervention: health education irrelevant to HIV/AIDS;
  HIV/AIDS interventions without health education
- Setting: non-college settings
- Outcome: policy not at the national level and not in Beijing
- Time period: out of the timeframe 1985–2016

### Policy content analysis

Based on the variables of the Statute Coherently Structures of Implementation proposed by the Framework of the Implementation of Public Policy [10], this study designed the policy content checklist through formal discussion in our research group to describe the ability of health education policy to structure implementation from the following aspects:

1. Incorporation of adequate causal theory
2. Unambiguous policy directives
3. Sufficient resources
4. Integration within and among implementation institutions
5. Monitoring and evaluation

The structure ability of policy was reviewed by two independent researchers, and discrepancies were discussed until agreements were reached.

### Cross-sectional surveys

The current study used data from two cross-sectional surveys in 2006 and 2016, respectively, to evaluate the target group-adolescent college students’ perceived impact of policy outputs and the actual impacts of policy outputs. Respondents included freshmen and sophomores as of 2006 and 2016 who registered at colleges in Beijing, People’s Republic of China. The 2006 data was selected from the subset of the Research on Health Education and Behavioral Intervention for HIV/AIDS Prevention among College Students funded by the Ministry of Health (MOH) of the People’s Republic of China, with respondents who were first-year and second-year students registered at colleges located in Beijing. The 2016 data were obtained from the survey using the same original questionnaire as the 2006 study—Questionnaire on AIDS Prevention Knowledge and Ability of College Students [12]. For reliability test, 70% of questions in the questionnaire reached over 0.4 for the Spearman rank correlation coefficient. For validity test, most of the questions reached over 0.6 for the Cronbach α coefficient.

### Measurement

Socio-demographic characteristics such as age, gender, and major were measured. For dependent variables, two sets of questions were asked. The first set of questions included the following:

1. Have your affiliated colleges ever conduct HIV/AIDS health education?
2. If yes in (1), HIV/AIDS health education was conducted by which department(s) in your college?
3. If yes in (1), what are the main means of HIV/AIDS health education in your colleges?

This study used “perceived HIV/AIDS health education” throughout the article to define HIV/AIDS health education conducted in the college which is aware by the students as measured by the first set of questions.

The second set of questions was designed to assess college students’ comprehensive knowledge of the essential facts about HIV transmission using UNGASS (United Nations General Assembly Special Session on HIV and AIDS) indicators (UNAIDS, 2009). The higher the score achieved, the better command of their knowledge.

1. Can the risk of HIV transmission be reduced by having sex with only one faithful, uninfected partner?
2. Can the risk of HIV transmission be reduced by using condoms?
3. Can a healthy-looking person have HIV?
4. Can a person get HIV from mosquito bites?
5. Can a person get HIV by sharing a meal with someone who is infected?

### Statistical analysis

All statistical analyses were performed using SPSS13©. The sample was described using frequency counts and percentages for categorical variables and means and standard deviations (SD) for continuous variables. *T* test, *χ*^2^ test, and logistic regression were used to analyze college students’ perception of HIV/AIDS education provided in their colleges and their knowledge of HIV/AIDS and their risk factors. For logistic analysis, study year, gender, and major were independent variables in the analysis of students’ perceived HIV/AIDS health education. While for the analysis of correctly answering UNGASS questions, study year, gender, major, and HIV/AIDS health education before college and at college were independent variables. A *P* value of less than 0.05 (two-tailed) was considered statistically significant.

## Results

In total, 14 pieces of national policy and 4 pieces of Beijing’s policy on college-based HIV/AIDS health education among college students were identified (Tables 1 and 2). The first piece of national policy on college-based HIV/AIDS health education was issued in 1998 and followed by the first Beijing’s policy in 2002. There was a 1-year’s lag of issuing municipal policy in Beijing after the national policy. The pre-2006 period witnessed the introduction of more than 60% of relevant policies. Numbers of policies introduced by each year peaked at 3 in both 2001 and 2004.

**Table 1:** Policy structure ability of national HIV/AIDS health education policy

Code	Issue date	Title	Issue body	Incorporation of adequate causal theory	Unambiguous policy directives	Resources availability	Integration between actors	M&E
Doc-1	November 12, 1998	China’s medium- and long-term plan for the prevention and control of AIDS (1998–2010)	MOH, SDPC, MOST, MOF	a	a	b	d	c
Doc-2	January 5, 2001	A notice on implementation guidance for China’s medium and long term plan for the prevention and control of AIDS (1998–2010)	MOH, SDOC, MOST, MOE, MOPS, MOJ, MOF, NRTA	a	a	b	b	a
Doc-3	May 25, 2001	China’s action plan for the prevention and control of AIDS (2001–2005)	GOSC	a	a	d	b	a
Doc-4	October 10, 2001	Ministry of Education’s opinion to carry out and implement the China’s action plan for the prevention and control of AIDS (2001–2005)	MOE	a	a	a	b	-
Doc-5	May 18, 2002	A notice on strengthening schools’ AIDS prevention efforts	MOE, MOH	a	a	b	b	d
Doc-6	March 16, 2004	State Council’s notice on effectively strengthening the work of AIDS prevention and treatment	State Council	b	c	b	b	a
Doc-7	April 15, 2004	National AIDS prevention and treatment publicity and education work guidance program(2004–2008)	SCAWC	a	a	b	b	b
Doc-8	May 19, 2004	Ministry of Education’s opinion to carry out and implement the State Council’s notice on earnestly strengthening the work of AIDS prevention and treatment	MOE	a	a	a	a	a
Doc-9	January 29, 2006	Regulation on AIDS prevention and treatment	State Council	a	a	c	b	a
Doc-10	February 27, 2006	China’s action plan for the prevention and treatment of AIDS (2006–2010)	GOSC	a	a	c	c	a
Doc-11	December 31, 2010	State Council’s notice on further strengthening the work of AIDS prevention and treatment	State Council	a	a	c	d	-
Doc-12	May 11, 2011	Ministry of Education and Ministry of Health’s opinion on further strengthening the prevention of AIDS in schools	MOE,MOH	a	a	a	c	a
Doc-13	January 13, 2012	China’s 12th 5-year action plan for the prevention and control of AIDS	GOSC	a	a	c	c	a
Doc-14	July 15, 2015	A notice on establishing an epidemic notification system to further strengthen HIV prevention and control in schools	GOHFPC, GOMOE	a	a	a	a	a

*MOH* Ministry of Health, *SDPC* State Development Planning Commission, *MOST* Ministry of Science and Technology, *MOF* Ministry of Finance. *MOPS* Ministry of Public Security, *MOJ* Ministry of Justice, *NRTA* National Radio and Television Administration, *GOSC*: General Office of the State Council, *SCAWC* State Council AIDS Working Committee, *GOHFPC* General Office of the Health and Family Planning Commission, *GOMOE* General Office of the Ministry of Education

^a^ represents high, ^b^ represents moderate, ^c^ represents low, ^d^ represents very low

**Table 2:** Analysis of Beijing policy’s ability to structure implementation

Code	Issue date	Title	Issue body	Incorporation of adequate causal theory	Unambiguous policy directives	Resources availability	Integration between actors	M&E
Doc-15	February 9, 2003	Notice of the General Office of the Beijing Municipal People’s Government on the issuance of the implementation program of the Beijing action plan on prevention and control of AIDS (2003–2005)	The General Office of the Beijing Municipal People’s Government	a	a	c	c	a
Doc-16	April 10, 2007	Beijing Education Committee’s notice to strengthen the health education work on prevention of AIDS in schools	Beijing Municipal Commission of Education	a	a	a	a	a
Doc-17	October 10, 2011	Notice of Beijing Municipal People’s Government on further strengthening AIDS prevention and treatment	The General Office of the Beijing Municipal People’s Government	a	a	c	a	a
Doc-18	August 8, 2012	Beijing’s 12th 5-year plan for AIDS control and prevention	The General Office of the Beijing Municipal People’s Government	a	a	c	a	b

^a^ represents high, ^b^ represents moderate, ^c^ represents low, d represents very low.

### Ability of policy structure implementation

To evaluate the structure ability of policy reviewed, a grading system of 5 categories was developed (see Table A1 in Appendix). For each component of policy ability to structure implementation, a grade (high, moderate, low, very low, or unclear) was assigned based on the evaluation of relevant policy content using the criteria pre-defined by the study group. Evaluation of national policy’s ability to structure implementation was summarized in Table 1. In general, national policy’s structure ability in the incorporation of adequate causal theory and the unambiguous policy directives were comparatively higher than other components, especially than the resource availability and the integration between actors. National policy’s structure ability in the monitoring and evaluation remained at a moderate level. Similar trends were identified in the review of Beijing’s policy (Table 2).

### Policy evolution

China’s first national policy on HIV/AIDS was issued by four ministries in 1998 (doc-2 November 12, 1998)*.* This medium- and long-term plan and its following implementation guidance in 2001 (doc-2 January 5, 2001) together established China’s HIV/AIDS prevention and control system including college-based HIV/AIDS health education. In 2001, the State Council issued the first national 5-year action plan on HIV/AIDS health education among college students (doc-3 May 25, 2001), followed consistently by the other two 5-year action plans on HIV/ADIS prevention in 2006 (doc-10 February 27, 2006) and 2012 (doc-13 January 13, 2012), requiring institutions of higher learning to distribute health education prescriptions and publicity materials on HIV/AIDS prevention and to set up lectures on the same topic to freshmen. Following the first national 5-year action plan, the Ministry of Education (MOE) issued an opinion in the same year to carry out and implement the action plan, suggesting institutions of higher learning to set up lectures or use health education classes to educate students on HIV/AIDS prevention (doc-4 October 10, 2001). Next year, the MOE together with the MOH issued a notice on strengthening schools’ HIV/AIDS prevention efforts, which specified the education sector should include HIV/AIDS prevention into the school’s integral working plan and implement the teaching content and time of HIV/AIDS prevention education in each school section while the health sector should provide technical supports and services for the education sectors (doc-5 May 18, 2002). In 2004, the State Council issued a notice on effectively strengthening the work of HIV/AIDS prevention and treatment, urging the health sector to include HIV/AIDS prevention and treatment knowledge into the teaching plan of institutions of higher learning (doc-6 March 16, 2004).

Soon after this, the State Council AIDS Working Committee (SCAWC) started a national guidance program on publicity and education work in HIV/AIDS prevention and treatment (doc-7 April 15, 2004), setting up a framework of HIV/AIDS publicity and education at colleges with clear quantitative objectives and multiple channels. This nationally sustainably adopted framework included the following aspects in HIV/AIDS prevention at colleges: training for school doctors and health education teachers, distribution of health education prescriptions for freshman, offer specific lectures and integrated it into health education classes, exhibition of relevant reading materials at library and reading room, and publicity through campus propaganda columns. It indicated by 2005; 100% of colleges should conduct health education on HIV/AIDS prevention with at least 1 lesson per academic year. MOE also issued an opinion to carry out and implement the State Council’s notice on earnestly strengthening the work of HIV/AIDS prevention and treatment, which restated these objectives proposed by the SCAWC guidance program.

By the year of 2006, the State Council issued its first regulation on HIV/AIDS prevention and treatment, stipulating the legal liabilities of People’s Governments at all levels and the health sector while requiring the education management department to guide and supervise colleges to include HIV/AIDS prevention and treatment knowledge into relevant courses and carry out relevant education activities after classes but no legal liability was stipulated (doc-9 January 29, 2006).

In 2011, the MOE and the MOH together issued an opinion further strengthening the prevention of HIV/AIDS in schools (doc-12 May 11, 2011), setting targets on colleges’ conduction of HIV/AIDS health education of 100% and students’ master of comprehensive knowledge on HIV/AIDS prevention and treatment of 90% by the year of 2015 (doc-12 May 11, 2011). Four years later in 2015, the MOE and the NHFPC issued a notice on establishing an epidemic notification system to further strengthen HIV/HIV prevention and control in schools, stating that colleges should establish a working mechanism between the Dean’s Office, Student Office, Youth League Committee, School Infirmary, and other departments to jointly promote the prevention of HIV/AIDS education (doc-14 July 15, 2015).

### Adolescent college students’ perceived impact of policy outputs

There were 1800 and 2001 participants in 2006 and 2016. In 2006, 59.61% of participants were female and in 2016, and 39.65% of participants were female in 2016. Regarding major, 22.83% and 19.69% were medical students in 2006 and 2016, respectively. In total, 51.38% of adolescent college students reported there were HIV/AIDS education activities carried out by their afflicted colleges with 71.14% and 39.80% in 2006 and 2016, respectively. Binary logistics showed that adolescent college students participated in the survey in the year of 2016 had 0.34 times of odds of perceiving HIV/AIDS education carried out at their colleges than those in 2006 (95% CI 0.25–0.43, *P* < 0.001) after controlling the following factors: year of the survey (2006/2016), gender, and major, indicating the decline of perceiving of HIV/AIDS education in colleges by students during these 10 years. Compared with the targets set by the national policies (doc-7 April 15, 2004, and doc-12 May 11, 2011), which require 100% of colleges to provide HIV/AIDS education by 2005 and by 2015, there remained a huge gap.

For participants who reported there was HIV/AIDS education provided their afflicted college, school associations (e.g., Red Cross Society) accounted for nearly 40% as sponsors of these activities, and there was no significant difference between 2006 and 2016 (Table 3). Activities sponsored by the School Infirmary and the Dean’s Office had showed significant increases during these 10 years while the Student Office and the Youth League Committee showed great decrease during the same period. It is noteworthy that more than a quarter of students reported they had no idea about the sponsors of AIDS education in their colleges.

**Table 3:** Sponsors of HIV/AIDS health education perceived by adolescent college students (%)

Sponsors	2006	2016	Difference	Total	***χ***^**2**^	***P***
School Associations	37.23	40.60	3.37	38.86	2.798	0.094
School Infirmary	26.11	34.86	8.75	30.34	21.237	< 0.001
Unknown	24.88	27.54	2.66	26.16	2.145	0.143
Student Office	21.09	9.89	− 11.20	15.68	55.624	< 0.001
Youth League Committee	21.17	5.47	− 15.70	13.59	122.96	< 0.001
Dean’s Office	11.20	14.92	3.71	13.00	7.148	0.008
Others	2.14	0.88	− 1.26	1.53	2.544	0.111

In terms of the main channels of HIV/AIDS education perceived by students (Table 4), lecture, publicity material, and peer education ranked as top 3 ways, though they all experienced a significant decrease from 2006 to 2016. These 10 years witnessed elective course and compulsory course to play a growing role in providing HIV/AIDS education in colleges. In sum, more than 80% of students reported lecture or course as their main channels in receiving HIV/AIDS education while only 31.48% of students they received information on HIV/AIDS on the window and bulletin board in colleges, merely half of the target in 2005 of 70% (doc-7 April 15, 2004) and far below the 85% target in 2008 (doc-7 April 15, 2004).

**Table 4:** Main channels of HIV/AIDS health education in 2006 and 2016 (%)

Main channel	2006	2016	Difference	Total	***χ***^**2**^	***P***
Lecture	65.26	46.57	− 18.69	56.26	82.635	< 0.001
Elective course	17.95	28.58	10.64	23.07	37.106	< 0.001
Compulsory course	13.23	18.52	5.29	15.78	12.249	< 0.001
Lecture or Course	83.67	89.70	6.04	87.44	37.335	< 0.001
Publicity material	54.01	47.20	− 6.82	50.73	10.823	0.001
Peer education	45.00	20.30	− 24.69	33.10	160.304	< 0.001
Window and bulletin board	32.34	30.54	− 1.80	31.48	0.872	0.350
Others	1.16	2.05	0.89	1.59	2.982	0.084

### Actual impacts of policy outputs

In total, 50.00% and 40.42% of students interviewed in 2006 and 2016, respectively, had comprehensive HIV knowledge by correctly answering all 5 UNGASS questions (Table 5). Mean score of all 5 UNGASS questions in 2016 (4.20, 95% CI 4.14–4.25) was significantly lower than that in 2006 (4.00, 95% CI 3.96–4.04) (*F* = 34.709, *P* < 0.001), indicating lower awareness of essential facts about HIV transmission among students, far from reaching the target more than 90% of the students master the knowledge of comprehensive prevention and treatment of HIV/AIDS by 2015 (doc-12 May 11, 2011). Logistics analysis showed that after controlling other factors, students received HIV/AIDS education before college (OR = 1.39, *P* < 0.001) or at college (OR = 1.35, *P* < 0.001) had a higher OR of correctly answering all UNGASS questions.

**Table 5:** Adolescent college students’ HIV/AIDS knowledge level using UNGASS questions [*n* (%)]

UNGASS question	2006	2016	***χ***^**2**^	***P***	Total
**Q1**: Can the risk of HIV transmission be reduced by having sex with only one faithful, uninfected partner?	1703 (97.48)	2763 (93.12)	41.88	< 0.001	4466 (94.74)
**Q2**: Can the risk of HIV transmission be reduced by using condoms?	1626 (93.07)	2622 (88.37)	27.287	< 0.001	4248 (90.11)
**Q3**: Can a healthy-looking person have HIV?	1522 (87.17)	2603 (87.79)	0.388	0.533	4125 (87.56)
**Q4**: Can a person get HIV from mosquito bites?	1132 (64.76)	1669 (56.14)	33.907	< 0.001	2801 (59.33)
**Q5**: Can a person get HIV by sharing a meal with someone who is infected?	1570 (89.87)	2345 (79.06)	91.27	< 0.001	3915 (83.07)
**All correct**	900 (50.00)	1213 (40.42)	41.907	< 0.001	2113 (44.01)
**Mean of UNGASS score**	4.20	4.00	34.709*	< 0.001	4.07

*F value was provided to compare the mean difference between two groups

## Discussion

To the best of our knowledge, this is the first study to review college-based HIV/AIDS education policy in China and Beijing, to analyze the ability of policy to structure implementation, and to assess adolescent college students’ perceived and actual impact of policy outputs. In total, fourteen pieces of national policy and four pieces of Beijing’s policy were identified. Policy’s ability to structure implementation was at a moderate level. The percentage of students in Beijing who ever perceived HIV/ADIS education at colleges decreased from 71.14 to 39.80% and the percentage of students with comprehensive knowledge of HIV/AIDS dropped from 50.00% in 2006 to 40.42% in 2016.

Results of the policy review showed that the State Council had been playing an active role since 2001 in comprehensive policy on the prevention and control of HIV/AIDS, including action plans, regulation, and notices. It is noteworthy that the MOE issued an implementation plan subsequently and had been working closely with the MOH/NHFPC in issuing policy on the school-based HIV/AIDS prevention and control. The Beijing Municipal People’s Government also followed the steps of the State Council in issuing action plans for HIV/AIDS control and prevention since 2003. Overall, HIV/AIDS prevention and control at college drew comparative attention from the national and Beijing’s government. The education sector was the primary executive department in HIV/AIDS prevention and control in college with technical supports from the health sector.

Though there was no previous study exploring the ability of policy to structure implementation in college-based HIV/AIDS health education, He et al. [13] summarized the challenges encountered in conducting HIV/AIDS health education practices at colleges. Financial and human resource insufficient was identified as one of the major challenges, which was consistent with the results of policy content review. He et al. also pointed out that the engagement of professionals in college-based HIV/AIDS health education was not enough.

Li reported in 2007 that 58.4% of students had ever perceived health education on HIV/AIDS prevention at their colleges [14], which was lower than the 2006 rate of 71.14% in our study. As our sample was origin from college students in Beijing, the capital city of China, where higher policy compliance and better implementation might be observed than Li’s study with a national average level.

Adolescent college students’ awareness of HIV/AIDS transmission using the UNGASS indicators in this study shared similarities with previous research [15–18]: college students’ awareness of reducing risk of HIV transmission by having sex with one partner was the best among all five categories while their awareness of mosquito bites not as HIV transmission was the worst. A national study in 2004 showed that 29.8% of surveyed college students in China correctly answered all 5 UNGASS questions on HIV prevention and transmission [19]. Similar results were found in population-based surveys in low- and middle-income countries in 2012 that 24% of young women and 36% of young men responded correctly for five questions on HIV prevention and HIV transmission [20]. Compared with these, the current study indicated that adolescent college students in Beijing had a higher awareness of HIV prevention and transmission of 44%.

This study identified a decline of policy outputs, both in adolescent college students’ perceived policy outputs and actual impact of policy outputs in 2016 compared with those of 2006. Adolescent college students who participated in the 2016 survey had 0.34 times and 0.75 times of odds of perceiving HIV/AIDS health education at their colleges and correctly answering all UNGASS questions than those in 2006, respectively. This was coincided with the decline of policy introduction from 2006 afterwards, indicating the gradual erosion of policy support. The comparatively lower odds students perceived HIV/AIDS health education than that of correctly answering all UNGASS questions indicating though students’ perception of HIV/AIDS health education declined dramatically, their HIV knowledge declined comparatively slighter. This might be due to the changing information acquiring channel as in 2016; students might use the Internet and mobile phone to acquire relevant knowledge without taking part in the traditional HIV/AIDS health education.

Thus, this study advocates further policy development to consider social mobilization and the whole government approach. Social mobilization aims at raising awareness, motivating stakeholders, and advocating human and financial resources that need to be adopted through the internet, mass and new medias, and other traditional channels to reach college students, teachers and staffs, the government, and other stakeholders, such as civil society organizations. According to a 2018 survey [21], mobile phone has become the 1st expected channel of publicity and education by Chinese residents, indicating mobile phone-based new medias, such as the WeChat with 963 million active users by June 2017 [22], could be the best way for health education. Besides, the whole government approach should be employed to integrate action across sectors in future policy and system development in this area. In addition to the leadership of the education sector, the health sector should be involved to provide technical supports and the financial sector needs to participate in financing guarantees and innovations. Furthermore, monitoring and evaluation should also be put emphasis on. Ensuring that policymaking is informed by sound evidence on what works is essential to achieve key long-term objectives. Mechanism of monitoring and evaluation should be incorporated into policy documents, and a routine data collection system should also be established and maintained. Capacity building of monitoring and evaluation is also needed.

However, this study had some limitations. Firstly, due to time constrain, we only analyze policy’s ability to structure implementation, other sets of variables in the framework, including the tractability of the problem, non-statutory variables affecting implementation, and stages in the implementation process, were not included in the current study, which might limit the understanding of policy implementation. Secondly, this study only considers HIV/AIDS health education sponsored by departments in colleges without including college activities initiated by sponsors outside colleges such as CDCs, which might limit our understanding of the whole picture. However, activities initiated by sponsors outside colleges are generally temporary activities rather than regular ones so not including these activities might not have a great impact on our results. Lastly, this study did not employ methods such as interview and we suggest future research to use this approach to better understand policy implementation process.

## Conclusions

The college-based HIV/AIDS education had drawn considerable attention from the Chinese government while the policy ability to structure implementation was moderate and the policy outputs were far below the targets set by relevant policies. This study highlighted the importance of policy evaluation and the need for further research on HIV/AIDS education policy in China.

## Supplementary information

**Additional file 1:.** Policy structure ability grading. Table A1 Policy structure ability grading system.

## Data Availability

The dataset analyzed during the current study is available from the corresponding author on reasonable request.

## Abbreviations

AIDS: Acquired immunodeficiency syndrome
HIV: Human immunodeficiency virus
MOE: Ministry of Education
MOH: Ministry of Health
SCAWC: State Council AIDS Working Committee
STD: Sexual transmitted diseases
UN: United Nations
UNAIDS: Joint United Nations Programme on HIV/AIDS
UNGASS: United Nations General Assembly Special Session
WHO: World Health Organization
