# Supplementary material for: Evaluation of the college-based HIV/AIDS education policy in Beijing, China: a mixed method approach
Source: Environ Health Prev Med. 2020 Sep 10;25:50. doi: 10.1186/s12199-020-00890-5 (PMC7488098; doi:10.1186/s12199-020-00890-5)
Supplement: Supplementary file 1 — Additional file 1:. Policy structure ability grading. Table A1 Policy structure ability grading system. [file 12199_2020_890_MOESM1_ESM.doc]

**Appendix Policy structure ability grading**

To evaluate the structure ability of policy reviewed, a grading system of 5 categories was developed (Table A1)..

Table A1 Policy structure ability grading system

| **Structure ability** | ***High***  ********** | ***Moderate***  ********* | ***Low***  ******** | ***Very low***  ******* | ***Unclear***  ***-*** |
| --- | --- | --- | --- | --- | --- |
| **Incorporation of adequate causal theory** | strong causal link between objectives and policy interventions | moderate causal link between objectives and policy interventions | weak causal link between objectives and policy interventions | very weak causal link between objectives and policy interventions | no causal link identified |
| **Unambiguous policy directives** | objectives are precise and clear ranked | objectives are precise but not clear ranked | objectives are clear but not precise | objectives are not clear and not precise | aims only, no objective identified |
| **Resources availability** | specific resources for school health education | specific resources for health education | resources guaranteed with considerations for health education | resources with considerations for health education | health education not mentioned in resources |
| **Integration between actors** | high extent of integration | integration between actors identified | multi-actor's roles and responsibilities defined | mentioned cooperation between different actors | not mentioned cooperation or integration |
| **Monitoring and Evaluation** | well-designed M&E system with core indicators | M&E system designed without indicators | M&E mechanism established | mentioned M&E only | not mentioned M&E |
